# Supplementary material for: Identification of Clinical Isolates of Candida albicans with Increased Fitness in Colonization of the Murine Gut
Source: J Fungi (Basel). 2021 Aug 27;7(9):695. doi: 10.3390/jof7090695 (PMC8468482; doi:10.3390/jof7090695)
Supplement: Supplementary file 1 [file jof-07-00695-s001.zip › jof-1337912-supplementary.pdf]

Supplementary Figure S1

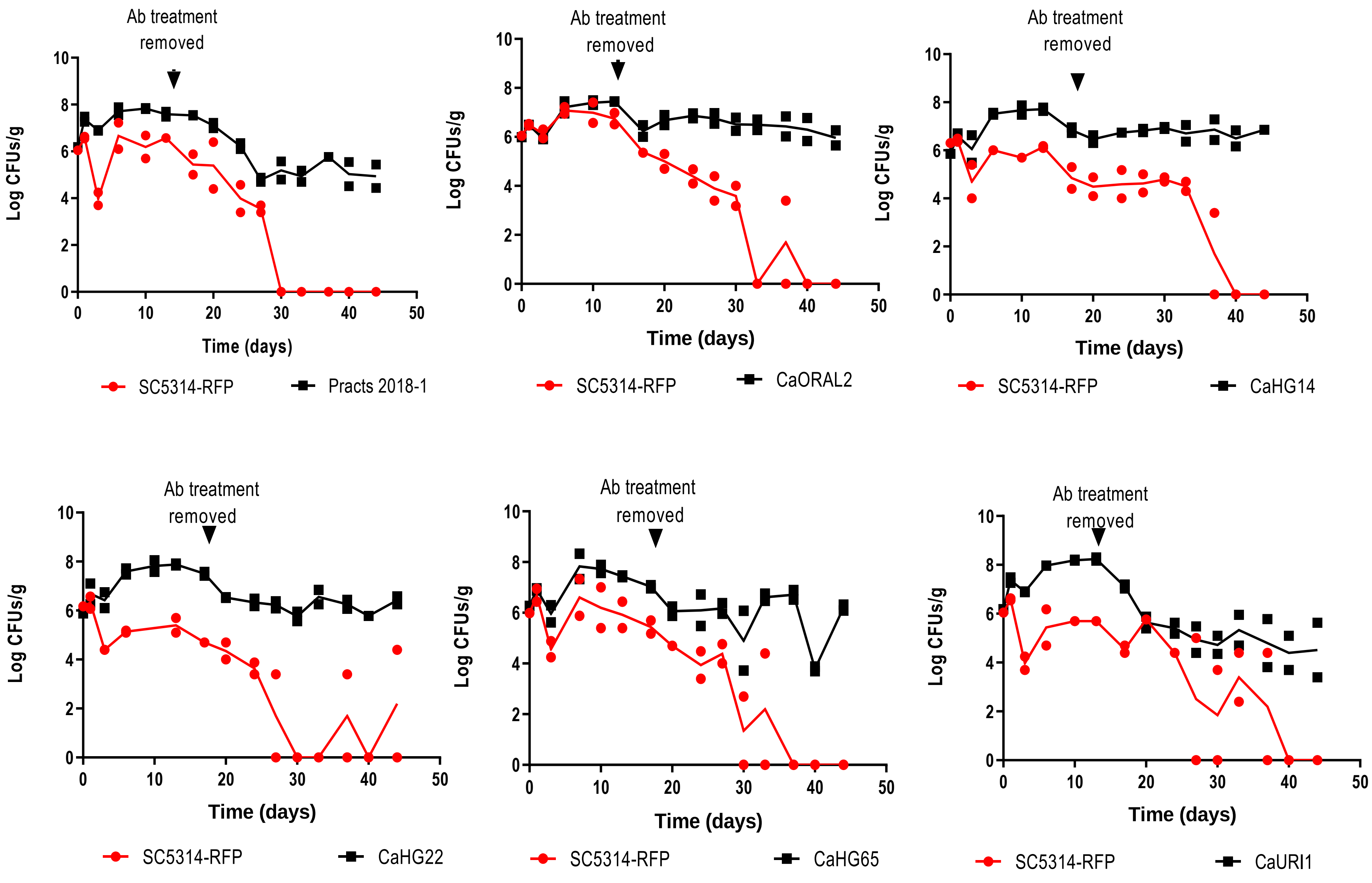

Supplementary Figure S2

24ºC
96 h

Phloxine B YPD plates

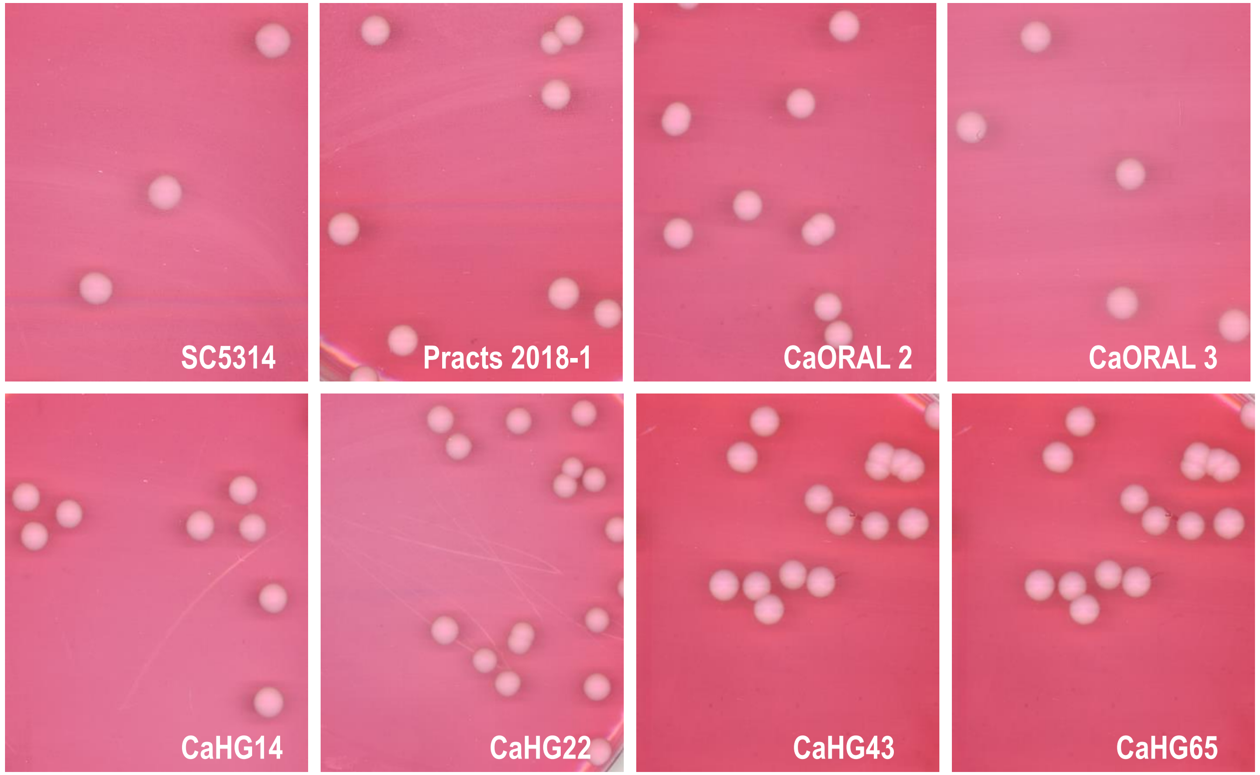

Supplementary Figure S3

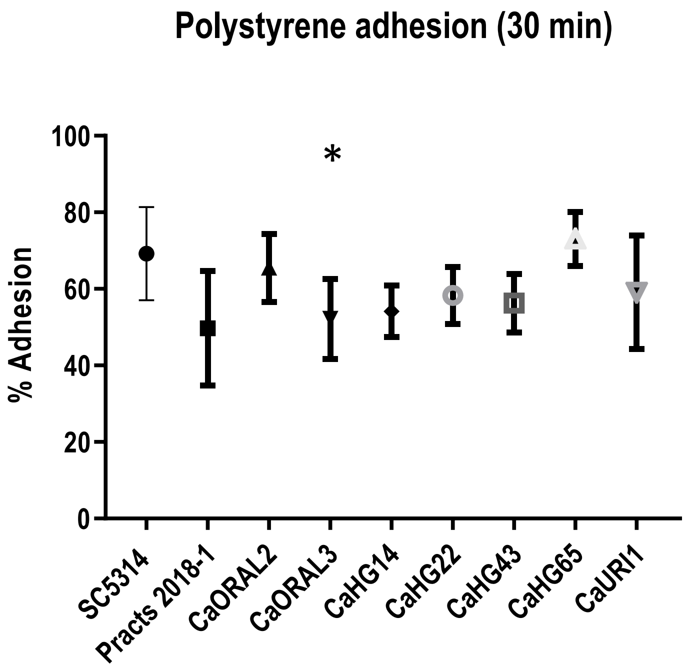

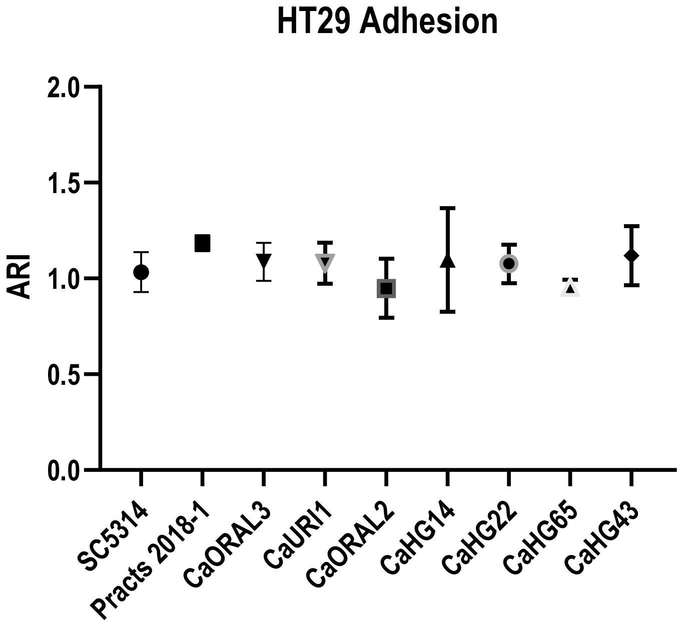

Supplementary Figure S4

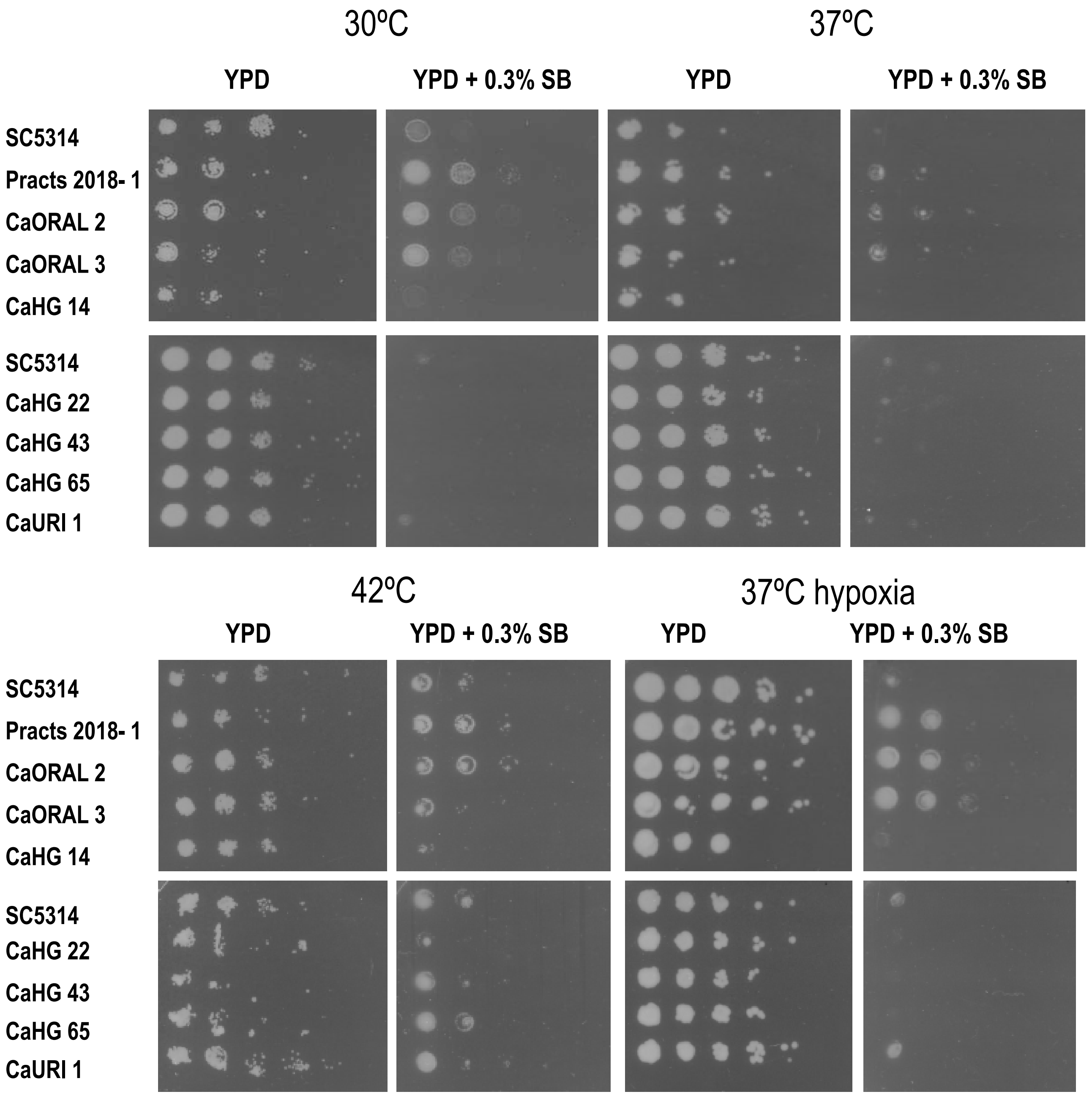

Supplementary Figure S5

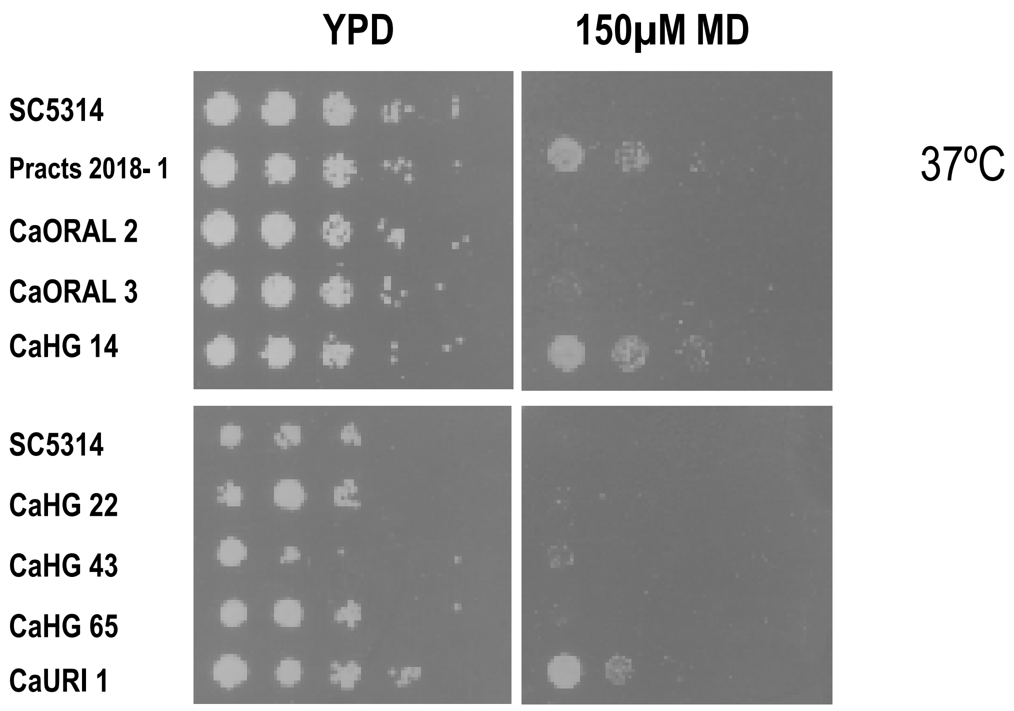

Supplementary Figure S6

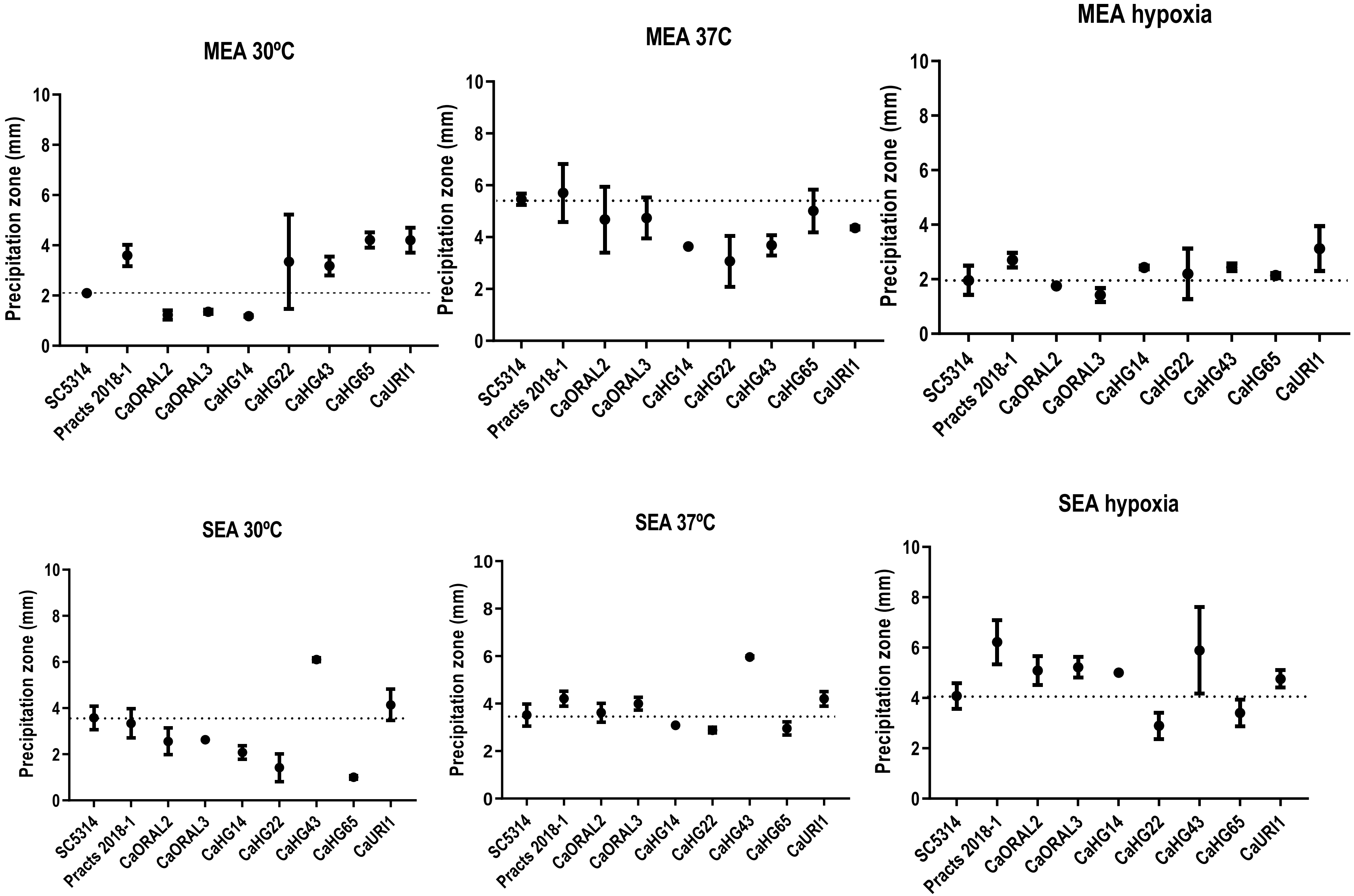

Supplementary Table S1

	MICs								
	SC5314	Practs2018-1	CaORAL2	CaORAL3	CaHG14	CaHG22	CaHG43	CaHG65	CaURI1
Caspofungin	0.06	0.06	0.06	0.03	0.06	0.06	0.06	0.06	0.015
Anidulafungin	0.06	0.06	0.12	0.03	0.12	0.12	0.06	0.03	≤0.015
Micafungin	≤0.08	0.015	0.015	≤0.08	≤0.06	≤0.06	≤0.08	≤0.08	≤0.08
5-Fluorocytosine	≤0.06	≤0.06	≤0.06	≤0.06	≤0.06	≤0.06	0.12	≤0.06	≤0.06
Amphotericin B	0.5	1	1	0.25	0.5	0.5	0.5	0.5	0.5
Itraconazole	0.03	0.06	0.06	≤0.015	0.06	0.06	0.06	0.03	0.03
Posaconazole	0.015	0.03	0.015	≤0.008	0.03	0.03	0.03	0.015	0.015
Fluconazole	0.25	0.5	0.25	0.25	0.5	0.5	0.5	0.25	0.25
Voriconazole	≤0.008	0.015	≤0.008	≤0.008	≤0.008	0.015	≤0.008	≤0.008	≤0.008
